# Advances in the intrinsic signaling pathway interactions and clinical translation of HR+/HER2+ breast cancer

**DOI:** 10.1007/s12282-025-01779-3

**Published:** 2025-09-22

**Authors:** Xinyu Li, Tao Huang

**Affiliations:** Department of Breast and Thyroid Surgery, Union Hospital, Tongji Medical College, Huazhong University of Science and Technology, 1277 Jiefang Rd., Wuhan, 430022 People’s Republic of China

**Keywords:** Breast cancer, HER2, Estrogen receptor, Target therapy, Endocrine therapy, Crosstalk

## Abstract

Hormone receptor-positive (HR +) and HER2-positive (HER2+) breast cancers represent a biologically unique subset of breast malignancies characterized by the co-expression of estrogen receptors (ER), progesterone receptors (PR), and human epidermal growth factor receptor 2 (HER2). These cancers exhibit distinct molecular features, often leading to aggressive growth and higher recurrence rates. HR+/HER2+ breast cancer cells can utilize estrogen and HER2-driven signaling pathways to promote proliferation, survival, and metastatic potential, presenting unique challenges and opportunities for treatment. The current therapeutic strategies focus on a combination of endocrine therapies, such as selective estrogen receptor modulators (e.g., tamoxifen) or aromatase inhibitors, with HER2-targeted therapies like trastuzumab, pertuzumab, or tyrosine kinase inhibitors, to concurrently inhibit both hormone and HER2-driven pathways. Despite initial treatment efficacy, resistance often develops through various mechanisms, including mutations in the PIK3CA gene, cross-talk between ER and HER2 signaling, and activation of alternative growth pathways. Ongoing research aims to improve patient outcomes by exploring novel combination therapies, including CDK4/6 inhibitors and PI3K/AKT/mTOR pathway inhibitors, and by targeting resistance pathways. This review highlights the molecular basis, treatment approaches, and emerging therapeutic strategies in HR+/HER2+ breast cancer, emphasizing the need for personalized and adaptive treatment strategies in managing this complex disease subtype.

## Introduction

Estrogen receptor (ER), progesterone receptor (PR), and human epidermal growth factor receptor 2 (HER2) are three key biomarkers used to classify breast cancer into four major molecular subtypes based on their expression levels [1]. The Luminal A and Luminal B subtypes, characterized by high hormone receptor expression, are typically responsive to hormone therapies that target the ER/PR signaling pathway. In contrast, the HER2-enriched subtype can be effectively treated with HER2-targeted therapies. Notably, HR-positive/HER2-positive breast cancer represents a distinct category due to the co-expression of ER (with or without PR) and HER2, differing from classical HER2-enriched or Luminal subtypes.

HR+/HER2+ breast cancer accounts for approximately 10–11% of all BC cases, representing 15% of Luminal BC and 70% of HER2-positive BC [2–4]. In addition to distinct biological features, HR+/HER2+ tumors exhibit unique clinicopathological characteristics compared with other subtypes. For instance, patients with HR+/HER2+ BC are more frequently diagnosed at a younger age and with more advanced disease [4]. According to the Surveillance, Epidemiology, and End Results (SEER) registry, the survival outcomes of HR+/HER2+ BC fall between those of HR+/HER2− and HR−/HER2+ subtypes, suggesting an intermediate prognosis [5]. Furthermore, the therapeutic response to HER2-targeted therapy differs by HR status, with lower efficacy observed in HR+ compared to HR− tumors [6, 7]. Notably, in both neoadjuvant and metastatic settings, the overall response rate (ORR) is significantly higher in HR− patients, though this does not necessarily translate into a proportional survival benefit [6, 8]. Collectively, these findings underscore the high heterogeneity of HER2-positive BC, emphasizing that HR+/HER2+ disease should be considered a distinct clinical entity requiring tailored therapeutic strategies.

In this review, we summarize the crosstalk between HR, with particular focus on the estrogen receptor (ER) pathway axis, and HER2 downstream signaling pathways and elucidate how their interconnected circuitry contributes to drug resistance and subtype-specific biological differences. Furthermore, we explore the translational potential of preclinical findings, aiming to bridge the gap between mechanistic insights and clinical applications in breast cancer therapy.

## Overview of HER2 and ER signaling pathway

### HER2 signaling pathway

The HER2 (human epidermal growth factor receptor 2) signaling pathway is a critical regulator of cellular proliferation, survival, and differentiation that plays a pivotal role in breast cancer pathogenesis. As a member of the ErbB receptor tyrosine kinase family comprising HER1 (EGFR/ErbB1), HER2 (ErbB2), HER3 (ErbB3), and HER4 (ErbB4), HER2 uniquely functions as a ligand-less receptor that serves as the preferred dimerization partner for other family members [9]. Structurally, these receptors contain an extracellular ligand-binding domain (except HER2), a single transmembrane α-helix, and an intracellular tyrosine kinase domain (non-functional in HER3) [9]. HER2 mediates signal transduction through dimerization, a process whereby two monomeric proteins from the EGFR family combine to form a dimeric complex. Specifically, this can occur through either homodimerization between two HER2 monomers (forming a HER2 homodimer) or heterodimerization between HER2 and other family members (EGFR/HER1, HER3, or HER4), which activates intrinsic tyrosine kinase activity, induces autophosphorylation, and recruits downstream adaptor proteins to initiate two major oncogenic pathways: the RAS/RAF/MEK/ERK cascade regulating cell proliferation and the PI3K/AKT/mTOR axis promoting cell survival [10]. HER2 gene amplification or overexpression leads to constitutive pathway activation that drives enhanced tumor cell proliferation, increased metastatic potential, and therapeutic resistance, making it a key therapeutic target in breast cancer [10].

### ER signaling pathways

The estrogen receptor (ER) signaling pathway serves as a fundamental regulator of mammary gland development and a key driver in hormone receptor-positive breast cancer pathogenesis. As a member of the nuclear receptor superfamily, ER exists in two primary isoforms: ERα (encoded by ESR1) and ERβ (encoded by ESR2), with ERα being the predominant and clinically relevant isoform in most hormone-positive breast cancers. Functioning as a ligand-activated transcription factor, ERα undergoes estrogen-induced conformational changes that facilitate dimerization and subsequent binding to estrogen response elements (EREs) in target gene promoters. Beyond this canonical genomic action, ERα can orchestrate transcriptional regulation through: (1) recruitment of co-activators (e.g., SRC family) or co-repressors to modulate ERE-dependent transcription; and (2) functional interplay with serum response factor (SRF) to indirectly regulate serum response element (SRE)-mediated gene expression. These mechanisms collectively activate networks governing cell cycle progression (e.g., Cyclin D1 upregulation), apoptosis inhibition (e.g., BCL2 induction), and growth factor signaling, ultimately promoting tumor cell proliferation and survival [11].

A subset of ERs is located near the cell membrane and interacts with other membrane proteins that initiates the non-canonical ER signaling pathway [12, 13]. The G-protein coupled estrogen receptor (GPER) is another membrane-associated receptor that also binds estrogen, synergized with membrane-associated ER to activate rapid kinase signaling cascade that modulate cell proliferation, apoptosis, cell migration and invasion (e.g. PI3K/AKT, MAPK/ERK and cAMP/PKA pathway). Estrogen binding to membrane-associated ERs can also trigger rapid changes in intracellular calcium levels, which contribute to signal transduction by activating calcium-dependent kinases (e.g. protein kinase C, PKC). In addition to estrogen-dependent activation, ER can also be activated through ligand-independent mechanisms by phosphorylation [11]. Growth factors such as epidermal growth factor (EGF) and insulin-like growth factor (IGF-1) activate receptor tyrosine kinases (e.g., EGFR, HER2), which in turn activate kinases like AKT and MAPK. These kinases phosphorylate ER at specific residues, resulting in ligand-independent activation of ER. Phosphorylated ER can then bind DNA and initiate transcription of estrogen-responsive genes, even in the absence of estrogen. This mechanism is especially relevant in breast cancer resistance to anti-estrogen therapies and lies the basis of crosstalk between ER and HER2 pathway.

### PR signaling pathway

The progesterone receptor (PR), also a member of the steroid nuclear receptor family, functions as both an ER-regulated gene product and a clinical biomarker for intact ER signaling [14]. Following progesterone binding, PR undergoes conformational changes that facilitate dimerization and nuclear translocation, enabling its binding to progesterone response elements (PREs) in target genes, with the PR-RANK-RANKL axis representing a crucial pathway in early mammary tumorigenesis [14, 15]. PR modulates ER activity through multiple mechanisms including direct physical interaction, chromatin binding site reprogramming, and regulation of ER-associated cofactors such as NRIP1, GATA3 and TLE3 [16, 17]. Similar to ER, PR exhibits both ligand-dependent and -independent activation, where in the latter case HER family receptor-mediated phosphorylation triggers non-genomic activation of PI3K/AKT and MAPK pathways to enhance cell survival signals [18]. Furthermore, PR regulates cell cycle progression by upregulating cyclin D1 through both direct transcriptional activation and formation of Stat3/ErbB-2 transcriptional complexes that drive cyclin D1 promoter activity, thereby promoting cellular proliferation [15, 19].

## Preclinical evidence of HER2 and HR pathway crosstalk

### Genetic and transcriptional activity crosstalk

The crosstalk between HER2 and estrogen receptor (ER) signaling pathways exhibits bidirectional regulation through multiple molecular mechanisms. First, HER2 directly phosphorylates both ER and its coregulators via its intrinsic tyrosine kinase domain, leading to ligand-independent hyperactivation of ER genomic signaling that enhances transcriptional activity and promotes expression of estrogen-responsive genes [20, 21]. Second, ER reciprocally modulates HER2 signaling by inducing phosphorylation of key cellular kinases including Src [22, 23] and PI3K [23, 24]. Furthermore, membrane-associated ER can directly interact with and activate the tyrosine kinase domain of HER2, initiating both direct and indirect stimulation of downstream effectors in the PI3K/AKT and MAPK/ERK pathways [13].

This bidirectional crosstalk is particularly facilitated by ER's non-genomic signaling, which establishes rapid communication with the HER2 pathway through an intricate protein interaction network. A central node in this network is the steroid receptor coactivator Amplified in Breast Cancer 1 (AIB1/SRC-3/NCOA3), which serves as a critical integration point between the two pathways. HER2-mediated post-translational modifications activate AIB1, which in turn phosphorylates and activates ER [25, 26]. Beyond this non-genomic regulation, AIB1 also bridges to ER genomic signaling by recruiting additional transcription factors and coactivators that collectively amplify ER target gene expression [27].

The synergistic crosstalk between HER2 and ER establishes a vicious cycle of mutual pathway activation that amplifies downstream oncogenic signaling while simultaneously developing compensatory resistance mechanisms to targeted therapies [28]. Although clinical data often show an inverse correlation between ER and HER2 expression, this relationship is dynamically modulated during treatment through genomic reprogramming that can downregulate both receptors, driving receptor-independent tumor progression [29]. Multiple studies indicate an inverse relationship between the expression of ER and HER2. Given the complex mechanisms of dual pathway feedback effects, it is important to note that receptor expression within tumors is dynamic and can change in response to targeted therapies [30]. Preclinical studies have shown that anti-HER2 treatment can induce adaptive transformations in cancer cells, leading to upregulated ER expression and acquired resistance to HER2-targeted therapies [31, 32]. Under conditions of excessive HER signaling activation, the activated downstream kinases downregulate ER expression while simultaneously promoting ER phosphorylation, thereby compromising the efficacy of endocrine therapy [33]. In triple-positive breast cancer (TPBC), both ER and HER2 signaling pathways remain active yet exhibit a reciprocal relationship. When one pathway dominates, the other becomes relatively suppressed. However, targeted therapy can trigger compensatory activation of the alternative pathway. This dynamic underlies the current research rationale for dual ER/HER2 targeted therapy in TPBC [34].

**Fig. 1:**
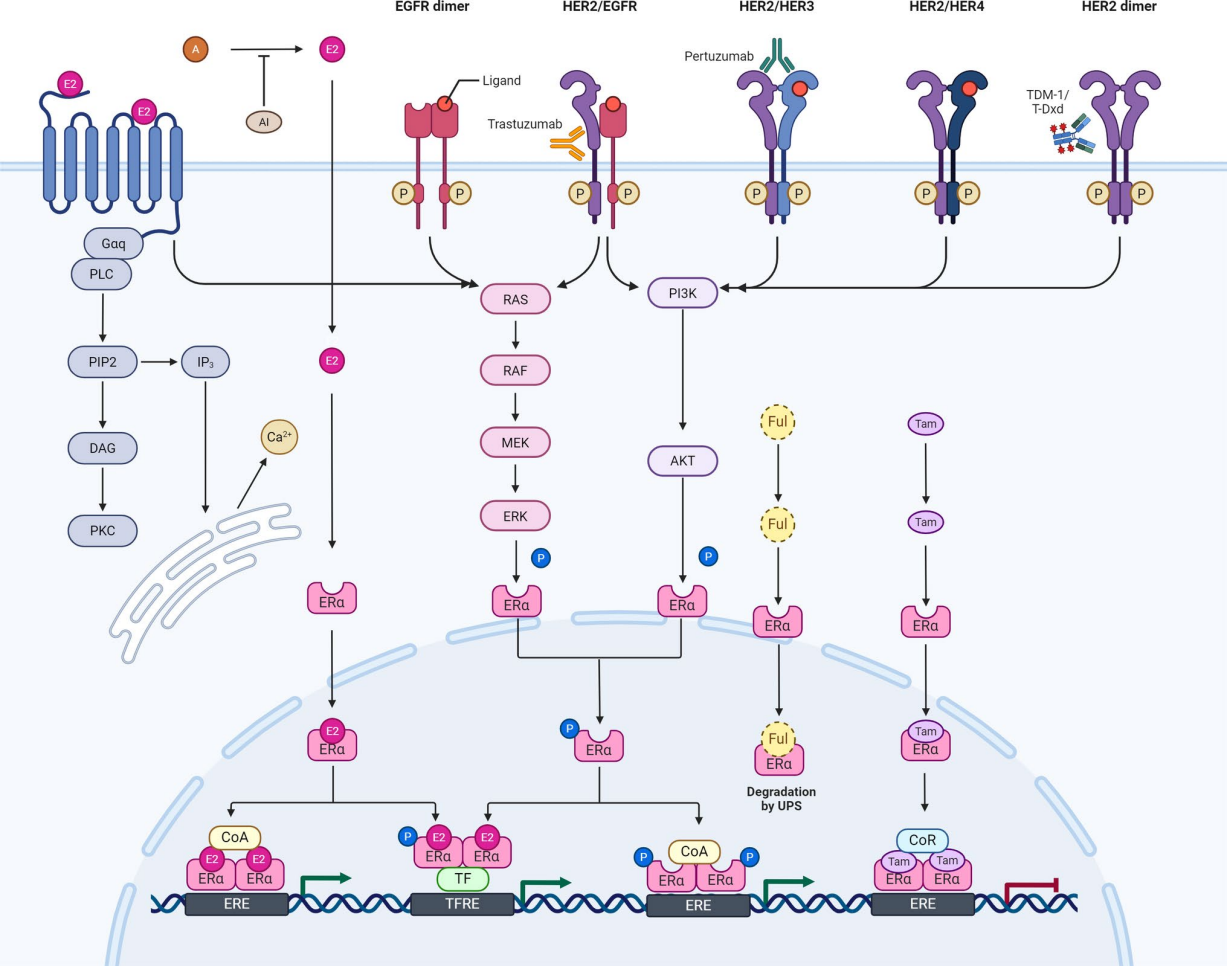
Crosstalk between the HER2 and ER signaling pathways

These bidirectional adaptations reflect the intrinsic heterogeneity of HR+/HER2+ tumors and underscore the clinical challenges posed by temporal and spatial heterogeneity. The emerging paradigm therefore necessitates combinatorial therapeutic strategies that concurrently target both pathways, coupled with longitudinal biomarker monitoring to track evolving receptor expression patterns during treatment [35] (Fig. 1).

### Tumor heterogeneity

Breast cancer heterogeneity manifests through two principal dimensions: inter-tumor heterogeneity (variations between patients) and intra-tumor heterogeneity (diversity within individual tumors) [36]. Inter-tumor heterogeneity stems from differential genetic mutations, epigenetic landscapes, gene expression profiles, and tumor microenvironment characteristics. The PAM50 classifier reveals important disparities between molecular subtypes and intrinsic biological classifications, with only 47% of HER2-positive breast cancers aligning with the HER2-enriched (HER2-E) subtype [37]. This distribution shows significant HR-status dependence, as HER2-E representation drops to 30% among HR+/HER2+ tumors [38]. Notably, PAM50 typically classifies HR+/HER2+ tumors as luminal subtypes, which demonstrate reduced sensitivity to anti-HER2 therapies compared to HER2-E tumors [39]. Meta-analyses confirm that the HER2-E subtype maintains consistent pathological complete response (pCR) rates across various neoadjuvant regimens, regardless of HR status [40]. Shen et al. identified that triple-positive breast cancer (TPBC) frequently exhibits Luminal A characteristics—marked by lower HER2 expression and favorable prognosis—and established biomarkers to identify TPBC subsets with luminal A-like features that show diminished response to anti-HER2 agents [41]. HR expression characteristics significantly influence tumor behavior and clinical outcomes, with both quantitative and qualitative analyses demonstrating prognostic importance [42, 43]. ASCO/CAP updated guideline stratifies HR expression into ER/PRlow (1–9%) and ER/PRhigh (≥ 10%) categories [44]. ER/PRlow HER2+ tumors show higher pCR rates than ER/PRhigh tumors, particularly with dual versus single anti-HER2 regimens [43, 45]. Paradoxically, while higher HR expression correlates with reduced pCR, it associates with improved long-term outcomes. Among ER +/HER2+ cases, PRhigh tumors demonstrate significantly better prognosis than PRlow or PR-negative counterparts [43]. This prognostic pattern persists in single HR-positive subgroups (ER +/PR- or ER-/PR +), which exhibit worse DFS and OS than TPBC [42, 46].

Intratumor heterogeneity could explain through two primary dimensions: spatial heterogeneity and temporal heterogeneity. It manifests as distinct cancer cell subpopulations within individual tumors that vary in gene expression profiles, protein levels, and receptor status [47, 48]. In ER +/HER2+ breast cancer, this heterogeneity is particularly evident in the differential expression patterns of ER and HER2 across tumor regions, with some cell populations showing high ER/low HER2 expression while others exhibit different profile [49]. Clinical sample evaluation showed that the presence of ER −/HER2+ and/or ER +/HER2- cell subpopulation has been independently associated with neoadjuvant treatment response [43, 49]. In addition, regional HER2 amplification variability affects treatment efficacy. The ASCO/CAP guidelines [50] define HER2 overexpression as immunohistochemistry (IHC) 3 + or IHC 2 + with amplification by in situ hybridization, where the proportion of IHC 2 + tumor cells in HER2+ patients is significantly associated with treatment response [51]. Moreover, spatial transcriptomics (ST) and single-cell RNA sequencing (scRNA-seq), as groundbreaking technologies in the biomedical field in recent years, have revolutionized the understanding of cellular heterogeneity. Yoshitake et al. [52] using ST and scRNA-seq technologies in ER + breast cancer, revealing intratumoral heterogeneity and identifying four spatially distinct functional compartments: estrogen-responsive (good prognosis), proliferative (drives growth, poor outcome), hypoxia-induced, and inflammatory—the latter two linked to aggressiveness and therapy resistance. Furthermore, these technologies enable the identification of drug-sensitive cell populations for targeted therapies. In HR+/HER2+ breast cancer, tumor cells from patients achieving pCR demonstrate spatial clustering characterized by high connectivity and short edge lengths, concurrent with a molecular subtype shift toward HER2-LUM (low hormone receptor expression). This distinct spatial architecture facilitates enhanced target-specific uptake of ADC therapeutics [53].

The co-existence of ER and HER2 signaling pathways within heterogeneous tumor populations creates a complex therapeutic challenge, as differential pathway activation across cellular subpopulations often leads to incomplete treatment response and disease relapse [54]. This biological heterogeneity manifests when HER2-targeted therapies eliminate HER2-overexpressing clones while allowing ER-driven subpopulations to persist, or conversely, when endocrine therapy fails to eradicate HER2-dependent cell fractions [55]. Such spatial variations in pathway dependence—where distinct tumor regions may predominantly utilize estrogen signaling, HER2 activation, or both mechanisms—underlie the reduced neoadjuvant therapy efficacy observed in HR+/HER2+ breast cancer [6]. Furthermore, these tumors demonstrate significant temporal heterogeneity, with receptor expression patterns evolving under treatment pressure [48, 56]. Following trastuzumab-based therapy, 20–50% of cases lose HER2 amplification, particularly in HR+ cohorts [57, 58], potentially developing more aggressive phenotypes [59]. It was also reported that high proportion of patients with residual disease observed loss of HER2 which highlight the uncertainty whether residual disease characterized by HER2 loss could still benefit from trastuzumab-emtansine (T-DM1, HER2-tageted antibody–drug conjugate) [60]. Similar dynamic alterations affect hormone receptors and these changes correlate with poorer survival outcomes compared to receptor-stable disease [54]. A small cohort study demonstrated that nearly 25% of the Luminal-HER2+ subtype showing changes in HR expression which was higher than Luminal-HER2- subtype [61]. These findings collectively highlight the critical need for ongoing receptor status monitoring and adaptive treatment strategies throughout the clinical course, as initial biomarker profiles may not accurately reflect the tumor's evolving biological state following therapy [62].

## Tumor microenvironment of HR+/HER2+ BC

The tumor microenvironment (TME) plays a pivotal role in the progression, therapeutic response, and resistance of HR+/HER2+ breast cancer (BC). As a complex and dynamic ecosystem, the TME comprises immune cells, fibroblasts, endothelial cells, extracellular matrix (ECM) components, and signaling molecules [63]. Immune modulation within the TME enables tumors to evade immune surveillance, adapt to targeted therapies, and develop resistance [64]. Tumor-infiltrating lymphocytes (TILs) serve as key indicators of the host's antitumor immune response and carry prognostic and predictive significance in HR+/HER2+ BC. For instance, the Neo-ALTTO trial demonstrated that baseline TIL levels independently predicted pCR and event-free survival (EFS) in HER2+ BC treated with anti-HER2 therapies [65]. However, emerging evidence suggests that dynamic changes in TILs during treatment may offer superior predictive value. The PAMELA study found that on-treatment TILs (measured after a 15-day window) correlated more strongly with pCR than baseline TILs [66]. In addition, multiple studies have linked pre- to post-neoadjuvant chemotherapy (NAC) TIL fluctuations with pCR rates, though elevated post-NAC TILs in residual disease may conversely reflect aggressive tumors with persistent immune evasion [67–69].

It is noteworthy that HR+/HER2+ tumors typically exhibit lower immune infiltration compared to more immunogenic subtypes like triple-negative BC (TNBC) or HR-/HER2+ disease [70]. Immune cell densities vary significantly by hormone receptor (HR) status, with HR- tumors showing higher infiltration than HR+ tumors. Furthermore, dual HER2 blockade markedly alters the immune contexture in HR- and HER2-enriched subtypes but not in HR+ or non-HER2-enriched subtypes [69]. Intriguingly, TILs may also predict tamoxifen (TAM) efficacy in ER + BC, with TAM demonstrating greater benefit in low-to-intermediate TIL environments [71].

Beyond TILs, distinct immune cell subsets and their functional signatures demonstrate comparable correlations with treatment response. Of particular significance, stromal-infiltrating T cells emerge as robust predictive biomarkers for both therapeutic response and clinical outcomes. The neoadjuvant TBCRC006 trial established that elevated baseline CD4 + T cell infiltration significantly correlated with improved pathological complete response (pCR) rates in patients receiving chemotherapy-free anti-HER2 regimens [72]. Substantiating these findings, Kenan et al. demonstrated that tumors exhibiting high CD8 + T cell density (≥ 25 cells) achieved pCR approximately four times more frequently than those with low CD8 + infiltration [73].

## Clinical implication for ER +/HER2+ breast cancer

### (neo)adjuvant therapy

Multiple prospective trials have demonstrated that HR+/HER2+ breast cancer exhibits lower pCR rates compared to HR-/HER2+ breast cancer under current HER2-targeted treatment strategies. In HR+/HER2+ patients receiving dual anti-HER2 therapy (e.g., trastuzumab + pertuzumab or TKIs), pCR rates range from 20 to 60%. For high-risk, early-stage HER2+ disease, taxane-based chemotherapy combined with dual HER2 blockade remains the cornerstone of treatment. Key trials, including NeoSphere [74], NeoALTTO [75] and PHEDRA [76], have reported an approximately 10% increase in pCR rates when pertuzumab or TKIs (lapatinib, pyrotinib) are added to trastuzumab-based chemotherapy, with ~ 40% of HR+ patients achieving pCR. Despite suboptimal neoadjuvant responses, HR+/HER2+ patients consistently exhibit favorable long-term survival outcomes [77–80]. Therefore, assessment of neoadjuvant therapy should incorporate not only pCR but also long-term survival benefits [81]. Personalized treatment strategies are imperative to minimize overtreatment, reduce toxicity, and optimize outcomes in biologically distinct subgroups.

Although trastuzumab-associated cardiotoxicity remains relatively uncommon, concurrent use with anthracyclines may potentiate cardiac dysfunction, exacerbating anthracycline-induced injury. Studies such as Cher-Lob [82], NSABP-B41 [83] and CALGB 40601 [84] have demonstrated comparable pCR rates with dual anti-HER2 therapy plus chemotherapy, irrespective of anthracycline inclusion. Furthermore, TRYPHAENA [85] and TRAIN-2 [86] evaluated anthracycline-free neoadjuvant regimens incorporating carboplatin, reporting similar pCR rates but improved tolerability, thus providing a viable alternative for anthracycline-ineligible patients. For biologically favorable cases, single-taxane regimens may also be considered [75].

Efforts to optimize treatment strategies have increasingly focused on de-escalating chemotherapy intensity to improve safety without compromising efficacy. Trastuzumab emtansine (T-DM1), an antibody–drug conjugate (ADC) combining trastuzumab with the cytotoxic payload DM1, reduces off-target toxicity by selectively delivering chemotherapy to HER2-expressing tumor cells. However, the PREDIX HER [87] and KRISTINE [88] trials demonstrated that T-DM1 (± pertuzumab) did not surpass standard chemotherapy combined with dual HER2 blockade in efficacy, with a numerial pCR advantage observed for chemotherapy-based regimens, particularly in the HR+ subgroup. The WSG-ADAPT-TP trial [80] evaluated a chemotherapy-free approach using T-DM1 plus trastuzumab and endocrine therapy (ET) in HR+/HER2+ disease, finding that T-DM1 outperformed trastuzumab monotherapy but did not derive additional benefit from ET. These findings underscore the need for careful patient selection when considering chemotherapy de-escalation.

Novel ADCs continue to reshape clinical paradigms. DESTINY-Breast11 is the first trial investigating trastuzumab deruxtecan (T-DXd) as neoadjuvant therapy for high-risk early HER2+ breast cancer, leveraging its unique ability to address intratumoral HER2 heterogeneity via a cleavable linker that enables bystander killing of low-HER2-expressing cells. Similarly, SHR-A1811, another HER2-targeted ADC structurally analogous to T-DXd, achieved a pCR rate of 63.3% in HER2+ patients in the FASCINATE-N trial [89], with HR+ patients attaining a pCR of 48.1% [53]. Notably, exploratory study found that non-pCR HR+ tumors exhibited elevated estrogen-related gene expression, suggesting a biological distinction in treatment resistance. Additionally, while HR+ tumors demonstrated lower immunogenicity, this did not correlate significantly with therapeutic response compared to HR- disease.

Further investigations into chemotherapy-free HER2 targeted regimens were explored in the TBCRC-006 [90] and TBCRC-023 [91] trials, which combined trastuzumab and lapatinib to achieve comprehensive HER family dimer blockade. In the ER + subgroup, only 21% of patients achieved pCR in TBCRC-006 trial but prolonged treatment duration (24 weeks vs 12 weeks) in TBCRC-023 trial improved pCR rates by 24%. The PAMELA trial [92] found that HER2-E intrinsic subtype benefited the most from dual HER2-blockade without chemotherapy, a finding corroborated by a joint analysis of these trials [93], which identified combined HER2-E subtype and ERBB2-high mRNA expression as predictive biomarkers for response and survival in the neoadjuvant setting.

Given preclinical evidence linking ER signaling to anti-HER2 resistance, concurrent blockade of both pathways was hypothesized to improve outcomes in HR+/HER2+ breast cancer. However, the Neo-ALL-IN trial [94], evaluating an all-oral regimen of aromatase inhibitors (AI) plus TKI, failed to demonstrate significant tumor regression, with no pCR observed. chemotherapy-free approaches in HR+/HER2+ patients typically yield pCR rates of 10–30%, even with biomarker stratification [95], and large-scale trials consistently report inferior outcomes compared to cytotoxic combinations [80, 96].

Dysregulation of the cyclin D1-CDK4/6-RB1 axis is a hallmark of ER + tumors [97], and CDK4/6 inhibitors have shown potential to reverse endocrine resistance and enhance HER2-targeted therapy efficacy in preclinical models [98, 99]. Despite this rationale, clinical translation remains exploratory, with most studies limited to small single-arm trials. The PALTAN trial [100] reported a disappointing pCR rate of 7.7% pCR with trastuzumab, letrozole, and palbociclib, whereas the MUDKEN-01 [101] and MUDKEN-01 Plus [34] trials observed more promising pCR rates with TKI + CDK4/6 inhibitor + ET ± trastuzumab in TPBC (without trastuzumab: 30.4%, with trastuzumab 58%)—a strategy warranting further validation. The NA-PHER2 trial [102] tested fulvestrant (a SERD drug) substitution for AI in a four-drug regimen, showing limited efficacy overall but suggesting potential utility in ESR1-mutated or AI-resistant populations. The TOUCH study [103] was the first achieved a pCR rate comparable to chemotherapy (32.9% vs. 33.3%) in postmenopausal patients using palbociclib + dual anti-HER2 therapy + letrozole, highlighting the feasibility of selective chemotherapy de-escalation.

Preclinical studies have demonstrated relatively high immunogenicity in HER2+ tumors, suggesting potential synergy with immune checkpoint inhibitors (ICIs). The Neo-Path trial [104] reported a pCR rate of 61% in the intention-to-treat population and 44% in HR+ patients with atezolizumab-containing regimens. Notably, PD-L1-positive patients achieved 100% pCR (13/13) versus 53% (28/53) in PD-L1-negative subgroups. However, the phase III IMpassion050 trial [105] failed to confirm these findings, showing no significant improvement with atezolizumab, even in PD-L1 positive populations. Importantly, ICI-containing regimens demonstrated poor tolerability, with high discontinuation rates [104].

Patients with residual disease following neoadjuvant therapy face an elevated risk of relapse, necessitating escalation to more intensive anti-HER2 regimens. The KATHERINE trial [106] established T-DM1 as the standard of care for this population, demonstrating superior invasive disease-free survival (iDFS) compared to dual HER2 blockade with trastuzumab and pertuzumab (HP). subgroup analyses revealed consistent iDFS benefits with T-DM1 regardless of HR status. Ongoing trials, including CompassHER2 RD (NCT04457596) and ASTEFANIA (NCT04873362) are evaluating novel strategies for high-risk residual disease, such as combining T-DM1 with tucatinib (a selective HER2/HER3 tyrosine kinase inhibitor [TKI]) or immunotherapy [107, 108]. Neratinib, an irreversible oral pan-HER TKI (targeting HER1, HER2 and HER4), has shown divergent efficacy across settings. In metastatic HER2+ breast cancer from NALA trial [109], neratinib + capecitabine did not improve outcomes in the HR+ subgroup (HR 1.08, 95% CI 0.84–1.40). Conversely, the ExteNET trial [110] demonstrated a 42% reduction in iDFS risk with neratinib in the adjuvant setting, particularly in HR+ patients.

In the adjuvant setting, 1-year trastuzumab has demonstrated long-term benefits, as evidenced by the HERA trial [111]. For high-risk patients—particularly those with lymph node metastasis, the addition of pertuzumab significantly improved DFS and OS in the APHINTY trial [112]. However, since HER2 BC patients are increasingly encouraged to undergo neoadjuvant therapy, those with low-risk tumors often proceed directly to post-surgical treatment. The 10-year follow-up data from the APT trial [113], which focused on stage I patients, confirmed that paclitaxel plus single-agent trastuzumab remains the standard of care for small, node-negative, HER2+ tumors. T-DM1 has also been investigated as a less toxic alternative to conventional chemotherapy in the adjuvant setting. Both the ATEMPT trial [114] and KAITLIN trial [115] reported comparable long-term outcomes between T-DM1 and traditional chemotherapy combined with HER2-targeted monoclonal antibodies. However, due to its high discontinuation rate, T-DM1 is primarily considered an alternative for patients who cannot tolerate standard chemotherapy.

Ultimately, individualized treatment strategies are critical, with an emphasis on de-escalating therapy—such as shorter durations or reduced-toxicity regimens—for patients with favorable prognostic features. Key trials are summarized in Table 1.

**Table 1:** Summary of pivotal clinical trial in early HER2-positive breast cancer

Trial	Eligibility	Population	Intervention	Study Endpoint	Safety	References
**Neoadjuvant therapy** Chemotherapy plus dual HER2 blockade						
**NeoSphere** (Phase II) NCT00545688	T > 2 cm, HER2+	HER + BC *N* = 417 HR+ subgroup *N* = 197	DH (*n* = 107) **vs** DHP (*n* = 107) **vs** HP (*n* = 107) **vs** DP (*n* = 96)	**ITT:** bpCR(ypT0/Tis) 29% vs 45.8% vs 16.8% vs 24%, pCR(ypT0/Tis,N0) 21.5% vs 39.3% vs 11.2% vs 17.7%, H vs HP: 5-year PFS 81% vs 86%, HR 0.69, 95% CI 0.34–1.40 **HR+ :** bpCR(ypT0/Tis) 20% vs 26% vs 5.9% vs 17.4%; H vs HP: 5-year PFS 87% vs 86%, HR 0.86, 95% CI 0.27–2.75	Serious AE 19% vs 14% vs 4% vs 17%	[74, 78]
**CherLob (**Phase II)	T > 2 cm, HER2+	HER + BC *N* = 121 HR+ subgroup *N* = 73	Pac-FEC plus Trastuzumab (*n* = 36) **vs** Laptinib (*n* = 39) **vs** Combination (*n* = 46)	**ITT:** pCR(ypT0/Tis,N0) 25% vs 26.3% vs 46.7% **HR+ :** pCR(ypT0/Tis,N0) 23.8% vs 20.8% vs 35.7%	Grade ≥ 3 AE Diarrhea 2.7% vs 33.3% vs 34.8%	[116]
**NSABP B-41** (Phase III)	T > 2 cm, HER2+	HER + BC *N* = 529 HR+ subgroup *N* = 331	AC- Paclitaxel plus Trastuzumab (*n* = 181) **vs** Laptinib (*n* = 174) **vs** Combination (*n* = 174)	**ITT:** bpCR(ypT0/Tis) 52.5% vs 53.2% vs 62%; pCR(ypT0/Tis,N0) 49.4% vs 47.4% vs 60.2%; ORR 76.8% vs 69.9% vs 82% **HR+ :** bpCR(ypT0/Tis) 46.7% vs 48% vs 55.6%; pCR(ypT0/Tis,N0) 45.5% vs 42% vs 54.6%	Grade ≥ 3 AE 4% vs 9% vs 9%, NYHA III/IV CHF 4% vs 4% vs 1%; Discontinuation 23% vs 35% vs 37%	[83]
**CALGB 40601** (Phase III) NCT00770809	T > 1 cm, Stage II ~ III, HER2+, non-multicentric	HER + BC *N* = 305 HR+ subgroup *N* = 176	Paclitaxel plus Trastuzumab (*n* = 118) **vs** Lapatinib (*n* = 64) **vs** Combination (HL) (*n* = 117	**ITT:** bpCR(ypT0/Tis) 46% vs 32% vs 56%; years RFS 79% vs 69% vs 93%; L vs H: HR 1.50, 95% CI 0.82–2.77, *P* = 0.191; HL vs H: HR 0.32,95% CI 0.14–0.71, *P* = 0.05 7-year RFS pCR vs RD, 89% vs 76%, HR 0.42, 95% CI 0.23–0.78, *P* = 0.006 7-year OS 88% vs 84% vs 96%; L vs H: HR 1.17, 95% CI 0.51–2.71, *P* = 0.711; HL vs H: HR 0.34,95% CI 0.12–0.94, *P* = 0.037 **HR+ :** bpCR(ypT0/Tis) 41% vs 29% vs 41%; 5-year EFS TH vs THL, 11/62 vs 6/60, HR 0.52, 95% CI 0.19–1.40, *P* = 0.188 7-year RFS pCR vs RD, 85% vs 77%, HR 0.67, 95% CI 0.32–1.41, *P* = 0.289	Serious AE 10.43% vs 13.04% vs 9.23%	[84]
**NeoALTTO** (Phase III) NCT00553358	T > 2 cm, HER2+	HER + BC *N* = 455 HR+ subgroup *N* = 232	Paclitaxel plus Trastuzumab (*n* = 149) vs Lapatinib (*n* = 154) **vs** Combination (HL) (*n* = 152)	**ITT:** bpCR(ypT0/Tis) 29.5% vs 24.7% vs 51.3%; pCR(ypT0/Tis,N0) 27.6% vs 20.0% vs 46.8%; ORR 70.5% vs 74.0% vs 80.2%; years EFS 64% vs 63% vs 67%; L vs H: HR 1.01, 95% CI 0.66–1.52, *P* = 0.98; HL vs H: HR 0.88,95% CI 0.57–1.34, *P* = 0.55 10-year OS 75% vs 76% vs 80%; L vs H: HR 0.96, 95% CI 0.58–1.60, *P* = 0.88; HL vs H: HR 0.79,95% CI 0.46–1.34, *P* = 0.38 **HR+ :** bpCR(ypT0/Tis) 22.7% vs 16.1% vs 41.6%; years EFS 67% vs 68% vs 69%; L vs H: HR 0.95,95% CI 0.51–1.75, *P* = 0.86; HL vs H: HR 0.85, 95% CI 0.46–1.58, *P* = 0.61 10-year OS 75% vs 77% vs 83%; L vs H: HR 0.93, 95% CI 0.44–1.92, *P* = 0.84; HL vs H: HR 0.63,95% CI 0.28–1.36, *P* = 0.24	Grade ≥ 3 AE Diarrhoea 2% vs 23.4% vs 21.1%, Hepatic 7.4% vs 18.1% vs 9.9%, Neutropenia 8.7% vs 15.6% vs 8.5%, Skin disorder 2.7% vs 6.5% vs 6.6% Discontinuation 8.1% vs 33.8% vs 39.5%	[75]
**PHEDRA (**Phase III) NCT03588091	T > 2 cm, HER2+	HER + BC *N* = 355 HR+ subgroup *N* = 195	Docetaxel and Trastuzumab plus Pyrotinib (*n* = 178) **vs** Placebo (*n* = 177)	**ITT:** pCR(ypT0/Tis,N0) 41% vs 22%; bpCR(ypT0/Tis) 43.8% vs 23.7%; ORR 91.6% vs 81.9% **HR+ :** pCR(ypT0/Tis,N0) 29.9% vs 12.2%	Grade ≥ 3 AE 71.3% vs 37.3, Diarrhea 44.4% vs 5.1%	[76]
**TRYPHAENA** (Phase II) NCT00976989	T > 2 cm, HER2+	HER + BC *N* = 225 HR+ subgroup *N* = 114	FECHP-THP (*n* = 73) **vs** FEC-THP (*n* = 75) **vs** TCbHP (*n* = 77)	**ITT:** bpCR(ypT0/Tis) 61.6% vs 57.3% vs 66.2%; pCR(ypT0/Tis,N0) 50.7% vs 45.3% vs 51.9%; CCR 50.7% vs 28% vs 40.3% **HR+ :** bpCR(ypT0/Tis) 61.6% vs 57.3% vs 66.2%, pCR(ypT0/Tis,N0) 46.2% vs 48.6% vs 50%	Grade ≥ 3 AE Neutropenia 47.2% vs 42.7% vs 46.1%, Leukopenia 19.4% vs 12% vs 11.8%, Diarrhea 4.2% vs 5.3% vs 11.8%; Serious AE 27.8% vs 20% vs 35.5%; Symptomatic LVSD 0% vs 4.0% vs 1.5%; LVEF decline (≥ 10%) 15.8% vs 22.9% vs 11.1%	[85]
**TRAIN-2** (Phase III) NCT01996267	Stage II ~ III, HER2+	HER + BC *N* = 438 HR+ subgroup *N* = 255	FEC-TCbHP (*n* = 219) **vs** TCbHP (*n* = 219)	**ITT:** pCR(ypT0/Tis,N0) 67% vs 68%, *P* = 0.95; **HR+ :** pCR(ypT0/Tis,N0) 51% vs 55%; OR 1.17, 95% CI 0.71–1.95	Serious AE 28% vs 22%, LVEF decline (≥ 10%) 29% vs 17%	[86]
Antibody–drug Conjugates						
**KRISTINE** (Phase III) NCT02131064	T > 2 cm, Stage II ~ III, HER2+	HER + BC *N* = 444 HR+ subgroup *N* = 277	Pertuzumab plus Trastuzumab emtansine (*n* = 223) **vs** Docetaxel, Carboplatin, Trastuzumab (*n* = 221)	**ITT:** pCR(ypT0/Tis,N0) 44.4% vs 55.7% 3-year iDFS 93% vs 92%; HR 1.11, 95% CI 0.52–2.4 **HR+ :** pCR(ypT0/Tis,N0) 46% vs 56% 3-year iDFS 92.2% vs 91.7%; HR 1.44, 95% CI 0.54–3.88	Grade ≥ 3 AE 13% vs 64%; Serious AE 5% vs 29%; Discontinuation 10% vs 8%	[88]
**PREDIX HER2** (Phase II) NCT02568839	T > 2 cm and/or node positive, HER2+	HER + BC *N* = 197 HR+ subgroup *N* = 125	Docetaxel, Trastuzumab and Pertuzumab (*n* = 99) **vs** Tratuzuma emtansine (*n* = 98)	**ITT:** pCR(ypT0/Tis,N0) 45.4% vs 43.9%; years EFS 89.6% vs 88.6%, HR = 1.26, 95% CI 0.54–2.91, *P* = 0.591; years RFS 91.6% vs 94.7%; years OS 96.7% vs 97.7% **HR+ :** pCR(ypT0/Tis,N0) 35.9% vs 34.6%	Grade ≥ 3 AE 68.7% vs 16.3%;	[87]
**HER2+ FASCINATE-N (**Phase II) NCT05582499	T2-3, N0-1, M0 or T2-3, N2-3, M0 or T4, any N, M0	HER + BC *N* = 60 HR+ subgroup *N* = 27	SHR-A1811	**ITT:** pCR(ypT0/Tis,N0) 63.3%; **HR+ :** pCR(ypT0/Tis,N0) 48.1%	-	
Immunotherapy						
**Neo-PATH** (Phase II) NCT03881878	Stage IIA ~ IIC, HER2+	HER + BC *N* = 67 HR+ subgroup *N* = 32	**Single arm:** Docetaxel, Trastuzumab, Pertuzumab and Atezolizumab	**ITT:** pCR(ypT0/Tis,N0) 61% **HR+ :** pCR(ypT0/Tis,N0) 44%	Grade ≥ 3 AE 31%; Atezolizumab withdrawal 11%	[104]
**Impassion050** (Phase III) NCT03726879	T > 2 cm with histologically confirmed positive lymph node, HER2+	HER + BC *N* = 454 HR+ subgroup *N* = 233	ddAC-PacPH plus Atezolizumab (*n* = 226) **vs** Placebo (*n* = 228)	**ITT:** pCR(ypT0/Tis,N0) 62.4% vs 62.7%; 1-year EFS 96.3% vs 97.9% **HR+ :** pCR(ypT0/Tis,N0) 50.9% vs 54.7%	Grade ≥ 3 AE 51.8% vs 43.6%; Serious AE 19.5% vs 13.3%; Discontinuation 14.2% vs 12.0%	[105]
HER2 blockade only (Chemo-free)						
**TBCRC 006** (Phase II) NCT00548184	T > 3 cm or T > 2 cm with palpable axillary lymph node, HER2+	HER + BC *N* = 64 ER + subgroup *N* = 39	**Single arm:** 12 weeks Lapatinib plus Trastuzumab(+ letrozole if ER +)	**ITT:** bpCR(ypT0/Tis) 27%; pCR(ypT0/Tis,N0) 22%; ypT1a-b 22% **ER + :** bpCR(ypT0/Tis) 21%; pCR(ypT0/Tis,N0) 18% ypT1a-b 33%	Discontinuation 6%; Grade ≥ 3 AE: Diarrhea 3%, ALT 5%, AST 6%	[90]
**TBCRC 023** (Phase II) NCT00999804	T ≥ 2 cm, HER2+	HER + BC *N* = 94 ER + subgroup *N* = 62	12 weeks Lapatinib plus Trastuzumab (*n* = 33) **vs** 24 weeks (*n* = 61) (+ letrozole if ER +)	**ITT:** bpCR(ypT0/Tis,N0) 12% vs 28%; Residual tumors ≤ 1 cm 21.2% vs 14.8% **ER + :** bpCR(ypT0/Tis,N0) 9% vs 33%; Residual tumors ≤ 1 cm 30% vs 13%	Grade ≥ 3 AE 6% vs 6%; Serious AE 3% vs 2%	[91]
**PAMELA (**Phase II) NCT01973660	T > 1 cm, Stage I ~ IIIA, HER2+	HER + BC *N* = 151 HR+ subgroup *N* = 77	**Single arm:** 18 weeks Lapatinib plus Trastuzumab(+ letrozole if ER +)	**ITT:** bpCR(ypT0/Tis) 30%, HER2-Enriched vs non-HER2-enriched 41% vs 10% **ER + :** bpCR(ypT0/Tis) 14%, HER2-Enriched vs non-HER2-enriched 33% vs 9%	Treatment failure 4%; Grade ≥ 3 AE: Diarrhoea 3%, Rash 2%	[92]
Co-target HR and HER2 in HR+/HER2+ BC						
**PerELISA** (Phase II) NCT02411344	Postmenopausal, ER + and/or PR + (> 10% cells), HER2+, Stage II-IIIA	HR+/HER + BC *N* = 61	Letrozole plus Trastuzumab and Pertuzumab (**Molecular responders** *n* = 44: Ki67 decrease > 20% after 2-week Letrozole) **vs** Paclitaxel plus Trastuzumab and Pertuzumab (**Molecular non-responder**s *n* = 17: Ki67 decrease < 20% after 2-week Letrozole)	pCR(ypT0/Tis,N0) 20.5% vs 81%; CRR 74% vs 94%; BCS rates 65.9% vs 63%, conversion to BCS rates 54.5% vs 57% **Molecular responders:** RCB-I 32%, RCB-II 62%, RCB-III 6%	Grade ≥ 3 AE: Molecular responders: Hypertension 2%, Muscoloskeletal symptoms 2%, Allergic reaction 2%; Molecular responders: Neuropathy 12%, Skin reactions 6%, Neutropenia 6%, Heart failure 6%, Abdominal pain 6%, GGT Increase 6%	[95]
**WSG-ADAPT-TP** (Phase II) NCT01779206	ER + and/or PR + (> 1% cells), HER2+	HR+/HER + BC *N* = 375	T-DM1 plus ET (*n* = 127) **vs** Trastuzumab plus ET (*n* = 129) **vs** T-DM1 (*n* = 119)	pCR(ypT0/Tis,N0) 41% vs 15.1% vs 41.5%; bpCR(ypT0/Tis) 43.4% vs 19.2% vs 42.4%; pCR(ypT0,N0) 34.1% vs 10.1% vs 32.5%; years IDFS 85.9% vs 84.6% vs 88.9%; HR 1.47, 95% CI 0.70–3.08, HR 1.57, 95% CI 0.75–3.30, *P* = 0.447; Years DDFS 95% vs 88.9% vs91.6%, HR 1.18, 95% CI 0.48–2.91, HR 1.58, 95% CI 0.66–3.75, *P* = 0.567; Years OS 96.4% vs 96.3% vs 97.2%, HR 1.38, 95% CI 0.42–4.58, HR 1.70, 95% CI 0.52–5.51, *P* = 0.671;	Grade ≥ 3 AE 7.5% in TDM-1 and TDM-1 + ET vs 4.1% in H + ET; Serious AE 5.3% vs 3.1%	[80]
**WSG-TP-II (**Phase II) NCT03272477	T > 1 cm, ER + and/or PR + (> 1% cells), HER2+ (IHC 3 + or 2 +/FISH +)	HR+/HER + BC *N* = 207	Endocrine plus Trastuzumab and Pertuzumab (*n* = 100) **vs** Paclitaxel plus Trastuzumab and Pertuzumab (*n* = 107)	pCR(ypT0/Tis,N0) 23.7% vs 56.4%; OR 0.24, 95% CI 0.12–0.46, *P* < 0.001 bpCR(ypT0/Tis) 26.8% vs 61.2%, *P* < 0.001 pCR(ypT0,N0) 10.3% vs 49.5%, *P* < 0.001 years EFS 92.1% vs 94.8%, HR 1.29, 95% CI 0.26–2.32, *P* = 0.65 Years OS 100% vs 97.9%	Serious AE 10% vs 12.1%; Cardiac Failure 1% vs 0%; Grade ≥ 3 AE 5% vs 28%,	[96]
**Neo-ALL-IN** (Phase II) NCT 01275859	Post-menopausal ER +/HER2+, Stage II ~ III BC	*N* = 24	Single arm: Letrozole plus Lapatinib	No pCR; RCB I 4.2%, RCB II 37.5%M RCB III 58.3%; Miller–Payne score: MP1 25%, MP2 33%, MP3 25%, MP4 8.3%, MP5 4.2%	Discontinue 29.2%; Grade ≥ 3 AE 45.9%	[94]
**PALTAN** (Phase II) NCT02907918	T > 2 cm, ER +, HER2+, Stage II-III	HR+/HER + BC *N* = 26	**Single arm:** Trastuzumab, Letrozole and Palbociclib	bpCR(ypT0/Tis) 7.7%; RCB-0 or RCB-1 38.5%, RCB-II 42.3%, RCB-III 19.2%	Grade ≥ 3 AE 73.1%; Serious AE 7.7%	[100]
**MUKDEN 01** (Phase II) NCT04486911	ER +, PR + and HER2+, Stage II-III	HR+/HER + BC *N* = 81	**Single arm:** Pyrotinib, Letrozole and Dalpiciclib	pCR(ypT0/Tis,N0) 30.4%; ORR 87.4%; bpCR(ypT0/Tis) 35.4%; RCB-0 or RCB-1 55.7%; Mean Ki67 reduces 40.4%(baseline) to 17.9%(surgery)	Grade ≥ 3 AE 68%, Neutropenia 53%, Leukopenia 20%, Diarrhea 17%	[101]
**MUKDEN 01 Plus** (Phase II) NCT05228951	ER +, PR + and HER2+, Stage II-III	HR+/HER + BC *N* = 12	**Single arm:** Trastuzumab, Pyrotinib, Letrozole and Dalpiciclib	pCR(ypT0/Tis,N0) 58%; RCB-0 or RCB-1 75%; ORR 92%; Mean Ki67 reduces 45.0%(baseline) to 17.2%(surgery)	Grade ≥ 3 AE 58%, Diarrhea 33%, Neutropenia 25%, Leukopenia 8%	[34]
**NA-PHER2** (Phase II) NCT02530424	T > 1 cm, HER2+ (3 + on IHC or neu-amplified) and ER + (> 10% cells)	HR+/HER + BC *N* = 30	**Single arm:** Trastuzumab, Pertuzumab, Palbociclib and Fulvestrant	ORR 97% (CR 50%, PR 47%) pCR(ypT0/Tis,N0) 27%	Grade ≥ 3 AE Diarrhoea 14%, Neutropenia 29%, Palbociclib discontinuation 17%	[102]
**TOUCH trial (**Phase II) NCT03644186	cT > 1 cm, cN0 or cN1, postmenopausal HR+/HER2+ BC	HR+/HER2+ BC *N* = 145	Trastuzumab,pertuzumab + Paclitaxel (T) (arm A = 73) **vs** Palbociclib plus Letrozole (arm B = 72)	pCR(ypT0/Tis,N0) 32.9% vs 33.3%	Grade ≥ 3 AE 31.5% vs 54.1%	[103]
**Adjuvant therapy**						
**APT (**Phase II) NCT00542451	≤ 3 cm, node-negative	HER + BC *N* = 406 HR+ subgroup *N* = 272	**Single arm:** Paclitaxel and Trastuzumab	**ITT:** 10-year IDFS 91.3%, 95% CI 88.3–94.4 10-year RFI 96.3%, 95% CI 94.3–98.3 **HR+ :** 91.6%, 95% CI 88.0–95.4 10-year RFI 96.2%, 95% CI 93.8–98.7	Grade ≥ 3 AE 14.78%; Cardiac disorder 3.2%	[113]
**HERA** (Phase III) NCT00045032	pN + or pN0 and > pT1c, HER2+	HER + BC *N* = 5099 HR+ subgroup *N* = 2571	2yrs or 1yrs of Trastuzumab (*n* = 1702) **vs** Observation (*n* = 1697) after adjuvant chemotherapy	**ITT:** 10-year DFS 69% vs 69% vs 63%; HR 0.77, 95% CI 0.69–0.87; HR 0.76, 95% CI 0.68–0.86 **HR+ :**10-year DFS 70% vs 72% vs 66%; (1-year trastuzumab vs observation) HR 0.77, 95% CI 0.69–0.87	Grade ≥ 3 AE 7.0% vs 4.4% Symptomatic congestive heart failure 1.73% vs 0.06% Decrease in LVEF 7.08% vs 2.21%	[30]
**APHINITY** (Phase III) NCT01358877	pN + or pN0 and high risk, HER2+	HER + BC *N* = 4805 HR+ subgroup *N* = 3082	Chemotherapy and Trastuzumab plus either Pertuzumab **vs** Placebo	**ITT:** iDFS HR 0.81, 95% CI 0.66–1.00, *p* = 0.045; 8-year iDFS 92.3% vs 90.6%; OS HR 0.83, 95% CI 0.68–1.02, *p* = 0.078; 8-year OS 92.7% vs 92.0% **HR+ :** iDFS HR 0.75, 95% CI 0.61–0.92; 8-year iDFS 88.9% vs 86.1%	Grade ≥ 3 AE 64.2% vs 57.3%; NYHA class III or IV heart failure and substantial decrease in LVEF 0.6% vs 0.2%	[112]
**ATEMPT (**Phase II) NCT01853748	Stage I(N0 or N1mi), HER2+	HER + BC *N* = 497 HR+ subgroup *N* = 373	TDM-1 (*n* = 383) **vs** Paclitaxel with Trastuzumab (*n* = 114)	**ITT:** 5-year iDFS 97.0% vs 91.1%; 5-year RFI 98.3% vs 93.2%; 5-year OS 97.8% vs 97.9% **HR+ : TDM-1** 5-year iDFS 96.8%, 95% CI 94.7–98.7;	CRTs 46% vs 47% Grade ≥ 3 AE 16% vs 23%; Symptomatic congestive heart failure 0.8% vs 1.8%; Declines in LVEF (≥ 15%) 0.5% vs 3.5%; Discontinuation 17% vs 6%	[114]
**KAITLIN** (Phase III) NCT01966471	pN ≥ 1 and any HR status or pN0, pT > 2 cm and ER/PR-negative, HER2+	HER + BC *N* = 1846 HR+ subgroup *N* = 1035	AC-THP (*n* = 918) **vs** AC-KP(TDM-1 + P) (*n* = 928)	**ITT:** iDFS HR 0.98, 95% CI 0.72–1.32; 3-year iDFS 94.2% vs 93.1%, HR 0.97, 95% CI 0.72–1.30; **HR+ :** 3-year iDFS 95.4% vs 94.1%, HR 0.94, 95% CI 0.61–1.44	Grade ≥ 3 AE 55.4% vs 51.8%; Serious AE 23.3% vs 21.4%; Discontinuation 26.8% vs 4%	[115]
**Post-neoadjuvant therapy**						
**KATHERINE (**Phase III) NCT01772472	T1–4, N0–3, M0 (excluding T1aN0 and T1bN0)] and non-pCR after ≥ 9 weeks of taxane/trastuzumab-based NAT	HER + BC *N* = 1486 HR+ subgroup *N* = 1074	TDM-1 (*n* = 743) **vs** Trastuzumab (*n* = 743)	**ITT:** 7-year IDFS 88.3% vs 77.0%; HR 0.54, 95% CI 0.44–0.66, *P* < 0.001; 7-year OS 89.1% vs 84.4%; HR 0.66, 95% CI 0.51–0.87, *P* = 0.0027 **HR+ :** 7-year IDFS 83.1% vs 69.8%; HR 0.52, 95% CI 0.40–0.67; 7-year OS 91.3% vs 85.9%; HR 0.60, 95% CI 0.42–0.85	Grade ≥ 3 AE 25.7% vs 15.4%; Discontinuation 18% vs 2.1%	[117]
**Extended therapy**						
**ExteNET** (Phase III) NCT00878709	Patients received adjuvant/neoadjuvant therapy with chemotherapy and trastuzumab	HER + BC *N* = 2840 HR+ subgroup *N* = 1631	Neratinib (*n* = 1420) **vs** Placebo (*n* = 1420)	**ITT:** 5-year IDFS 90.2% vs 97.7%; HR 0.73, 95% CI 0.57–0.92, *P* = 0.0083 years DFS 89.7% vs 86.8%; HR 0.71, 95% CI 0.56–0.89, *P* = 0.0035 Years DDFS 91.6% vs 89.9%; HR 0.78, 95% CI 0.60–1.01, *P* = 0.065 **HR+/≤ 1 years:** 5-year IDFS 90.8% vs 86.7%; HR 0.58, 95% CI 0.41–0.82, *P* = 0.002; Years DDFS 92.4% vs 87.7%; HR 0.57, 95% CI 0.39–0.83, *P* = 0.003; 8-year OS 91.5% vs 89,4%; HR 0.79, 95% CI 0.55–1.13, *P* = 0.203 **HR+/> 1 years:** 5-year IDFS 93.0% vs 91.7%; HR 0.74, 95% CI 0.29–1.84, *P* = 0.523	Serious AE 7% vs 6%; Grade ≥ 3 diarrhoea 40% vs 2%	[110]

The bold values is intended to highlight and differentiate the data between the Intention-to-Treat population and subgroup population

### Metastatic stage

For patients with late-stage, non-operable HER2-positive breast cancer (regardless of hormone receptor [HR] status), the standard first-line treatment consists of chemotherapy combined with dual anti-HER2 blockade. The CLEOPATRA trial [118] established the efficacy of taxane plus trastuzumab and pertuzumab in this setting, demonstrating a 31% reduction in the risk of death (HR 0.69, 95% CI 0.58–0.82) and a median overall survival (OS) of 57.1 months as compared to the placebo group. This survival benefit remained significant in the HR-positive subgroup (HR 0.74, 95% CI 0.58–0.96). Given the success of dual HER2 inhibition, the PHILA trial [119] evaluated trastuzumab plus docetaxel with or without pyrotinib (an irreversible tyrosine kinase inhibitor targeting HER1, HER2, and HER4). The addition of pyrotinib significantly prolonged median progression-free survival (PFS) compared to the control arm (24.3 vs. 10.4 months, HR 0.41, 95% CI 0.32–0.53, *p* < 0.001), with consistent benefit observed in the HR-positive subgroup (HR 0.47, 95% CI 0.35–0.65). The PFS improvement with pyrotinib was comparable to that seen with pertuzumab in the CLEOPATRA trial (mPFS 18.7 months).

Given their promising antitumor activity and favorable toxicity profiles, HER2-targeting antibody–drug conjugates (ADCs) have emerged as key therapeutic options in metastatic breast cancer (MBC) [120]. However, T-DM1 failed to demonstrate superiority over chemotherapy plus dual HER2 blockade (trastuzumab/pertuzumab) in the first-line setting, showing comparable PFS and OS outcomes without significant improvement [121]. Nevertheless, due to its improved safety profile, T-DM1 has become the standard second-line treatment for HER2+ MBC following trastuzumab failure, as evidenced by the EMILA trial [122] where it outperformed lapatinib plus capecitabine. T-Dxd has demonstrated remarkable efficacy in later-line treatment [123, 124]. The DESTINY-Breast02 [124] and DESTINY-Breast03 [125] trials showed that T-Dxd reduced the risk of progression or death by nearly 70% and improved OS by approximately 50% compared to standard therapies. In the second-line setting, T-Dxd exhibited superior efficacy over T-DM1, establishing it as a preferred alternative. While the PFS benefit of T-Dxd appeared somewhat attenuated HR+/HER2+ disease compared to HR-negative cases (DB-02: HR 0.41 vs 0.32; DB-03: HR 0.38 vs 0.34), the clinical benefit remained meaningful. Given T-Dxd’s enhanced activity in HER2-low disease [126] and its potential to overcome resistance mechanisms associated with HER2 expression changes [57], it represents an optimal option for third-line therapy following T-DM1 failure [124, 127].

For patients with brain metastasis, tucatinib has emerged as a pivotal therapeutic option. The HER2CLIMB trial [128] demonstrated that adding tucatinib to trastuzumab and capecitabin reduced the risk of intracranial progression or death by 68%, with ana intracranial ORR of 47.3%. These findings were corroborated in the HER2CLIMB-02 trial [129], where tucatinib combined with T-DM1 significantly improved PFS as compared to T-DM1 alone (ITT population: median PFS 9.5 vs 7.4 months, HR 0.76, *P* = 0.0163; Brain-metastasis subgroup: median PFS 7.8 vs 5.7 months, HR 0.64). Concordant benefits were observed in HR+ patients, offering a valuable new strategy for managing HER2+ MBC with central nervous system involvement.

For HR+/HER2+ breast cancer, the interplay between estrogen receptor (ER) and HER2 signaling pathways creates both therapeutic opportunities and challenges. Although cytotoxic chemotherapy remains the backbone of treatment for aggressive disease, endocrine-based strategies have emerged as viable options for selected patients without visceral crisis. This section examines the evolving evidence for chemo-free regimens in this molecular subtype. Preclinical models demonstrate that ER-HER2 crosstalk contributes to treatment resistance, suggesting that single-agent targeted therapy may be insufficient for durable disease control [130]. Although laboratory data support dual receptor blockade strategies, clinical evidence for this approach remains inconclusive [28, 32]. The French Epidemiological Strategy and Medical Economics (ESME) MBC database analysis (*N* = 4,145 HER2+ MBC, including 2,696 HR+ cases) found no significant differences in OS or PFS between chemotherapy and endocrine therapy plus anti-HER2 regimens. However, the limited sample size in the dual-HER2 blockade subgroup reduced the credibility of the study [131]. The TAnDEM trial [132] was the first to investigate the combination of single hormonal drug and trastuzumab without chemotherapy in HR+ HER + MBC as first-line treatment. The results showed that adding trastuzumab to anastrozole had better median PFS compared to anastrozole alone (mPFS 4.8 vs 2.4 months, HR 0.63, 95% CI 0.47–0.84). Same as the results of the eLEcTRA and the EGF 30008 studies, co-targeted regimens significantly enhanced the efficacy of estrogen deprivation [133, 134]. Use of dual HER2-targeted therapies appears more convincing which PERTAIN trial [135] demonstrated pertuzumab and trastuzumab plus AI significantly improved PFS compared to trastuzumab plus AI (mPFS 18.89 vs 15.80 months, HR 0.65, 95% CI 0.48–0.89), with a more pronounced benefit in patients who did not receive induction chemotherapy (mPFS 21.72 vs 12.45 months, HR 0.55, 95% CI 0.34–0.88). The same conclusions found with the addition of lapatinib to trastuzumab plus AI in the ALTERNATIVE trial [136], which showed a significant 38% reduction in the risk of disease progression (mPFS 5.6 vs 8.3 vs 11 months, HR 0.85, 95% CI 0.45–0.88). It is worth notice that these conclusions were built on postmenopausal population which their applicability to premenopausal women is uncertain. The PLEHERM trial [137] evaluated the efficacy of pyrotinib with letrozole in HR+/HER + MBC, yielding a clinical benefit rate (CBR) of 71.7% and the CBR was consistent among pre- and postmenopausal patients (CBR: 73.9% and 70%). Overall, ET combined with anti-HER2 agents has shown significant benefits in maintenance therapy. However, the use of ET as a substitute for chemotherapy remains controversial, primarily due to the lack of clinical evidence comparing the efficacy of both treatments in patients with manageable clinical risks. The SYSUCC-002 trial [138] provided the first head-to-head comparison, showing non-inferior PFS for ET + trastuzumab versus chemotherapy + trastuzumab (19.2 vs 14.8 months; HR 0.88, 95% CI 0.71–1.09). However, the absence of dual HER2 blockade in either arm limits applicability to current standards. Another chemo-free approach with dual HER2 blockade + ET showed shorter PFS than chemotherapy-based regimens in the HR+ cohort (mPFS 8.3 vs 25.5 months), though 2-year OS rates was comparable (76.5% vs 75.8%) [139]. Intriguingly, when all patients subsequently received second-line T-DM1 treatment, PFS was significantly better in the chemo-free group (mPFS 8.9 vs 6.4 months). These results indicate that ET combined with HER2-targeted therapy may represent a viable first-line treatment option for chemotherapy-intolerant patients or those with favorable prognostic characteristics, especially treatment-naïve patients with de novo advanced breast cancer who tend to respond better to targeted therapies.

Metastatic breast cancer often develops complex drug resistance mechanisms following multiple lines of targeted therapies. In HR+/HER2- advanced disease, pivotal trials including MONALEESA-2 [140] and MONARCH-3 [141] have established CDK4/6 inhibitors combined with endocrine therapy as the first-line standard. Preclinical evidence suggested that CDK4/6 inhibitors may overcome HER2-targeted therapy resistance [98], with clinical validation emerging from several trials. The PATRICIA trial [142] demonstrated activity of palbociclib(CDK4/6i) plus trastuzumab in postmenopausal, anti-HER2-pretreated patients, while the PATINA trial [143] showed significant PFS improvement (HR 0.74, 95% CI 0.58–0.94, *p* = 0.0074) when adding palbociclib to anti-HER2 therapy and endocrine therapy following chemotherapy induction in advanced TPBC patients. The MonarcHER trial [144] further supported this approach, revealing superior PFS and ORR (mPFS 8.3 vs 5.7 vs 5.7 months; ORR 36% vs 16% vs 16%) for CDK4/6 inhibitor combinations compared to standard chemotherapy plus trastuzumab. The DETECT V trial [145] found comparable efficacy between ribociclib-containing chemo-free regimens and chemotherapy-based approaches, suggesting CDK4/6 inhibitors may offer a viable first-line option. Ongoing investigations like the PLEASURABLE trial [146] evaluating all-oral regimens may further optimize treatment compliance through reduced toxicity. Details of studies regarding hormone therapy combined with HER2-targeted therapy were listed in Table 2.

**Table 2:** Emerging ER and HER2 co-targeted therapies for patients with advanced-stage HR-positive and HER2-postive breast cancer

Trial	Eligibility	Population	Intervention	Study Endpoint	Safety	References
**TAnDEM** (Phase III) NCT00022672	1–2 lines (Previous hormonal therapy for MBC was permitted; Prior chemo or anti-HER2 therapy was not permitted)	207 Postmenopausal only	Anastrozole with(*n* = 103) **vs** without Trastuzumab(*n* = 104)	mPFS 4.8 vs 2.4 months, HR 0.63, 95% CI (0.47–0.84), *P* = 0.0016 mTTP 4.8 vs 2.4 months, *P* = 0.0007 mOS 28.5 vs 23.9 months, *P* = 0.325 years OS 57% vs 50% CBR 42.7% vs 27.9% ORR 20.3% vs 6.8%	Grade ≥ 3 AE 23% vs 5% All grade cardiac AEs 13.6% vs 1.9% Symptomatic congestive heart failure 0.9% vs 0%; Declines in LVEF (≥ 15%) 0.9% vs 0%	[132]
**EGF 30008** (Phase III) NCT00073528	First-line	219 Postmenopausal only	Letrozole with(*n* = 111) **vs** without Lapatinib(*n* = 108)	mPFS 8.2 vs 3 months, HR 0.71, 95% CI (0.53–0.96), *P* = 0.019 mTTP 4.8 vs 2.4 months, *P* = 0.0007 mOS 28.5 vs 23.9 months, *P* = 0.325 years OS 57% vs 50% CBR 48% vs 29% ORR 28% vs 15%	Grade ≥ 3 AE 8% vs 4%; Declines in LVEF (≥ 15%) 0.8% vs 0.3%	[133]
**eLEcTRA** (Phase IV) NCT00171847	First-line	57 Postmenopausal only	Letrozole with(*n* = 26) **vs** without Trastuzumab(*n* = 31)	mTTP 3.3 vs 14.1 months, HR 0.67, 95% CI (0.35–1.29), *P* = 0.23 CBR 65% vs 39% ORR 27% vs 13%	Grade ≥ 3 AE 23% vs 5% All grade cardiac AEs 13.6% vs 1.9% Symptomatic congestive heart failure 0.9% vs 0%; Declines in LVEF (≥ 15%) 0.9% vs 0%	[134]
**PLEHERM** (Phase II) NCT04407988	First-line	53	**Single arm:** Pyrotinib plus Letrozole	mPFS 13.7 months; 1-year PFS 54.86% CBR 71.7%; ORR 64.2%	Grade ≥ 3 AE 18.9%	[137]
**SYSUCC-002** (Phase III) NCT01950182	First-line (Prior Trastuzumab disease free interval must > 12 months)	392	Trastuzumab plus Endocrine therapy(*n* = 196) **vs** Chemotherapy(*n* = 196)	mPFS 19.2 vs 14.8 months, HR 0.88, 95% CI (0.71–1.09), *P* < 0.0001 mOS 33.9 vs 32.5 months, 0.82, 95% CI (0.65–1.04), *P* = 0.094 CBR 94.9% vs 85.7% ORR 42.9% vs 37.2%	Grade ≥ 3 AE 3.1% vs 51.0%	[138]
**PERTAIN** (Phase II) NCT01491737	First-line	258 Postmenopausal only	Letrozole or Anastrozole plus Trastuzumab with(*n* = 129) **vs** without Pertuzumab(*n* = 129)	**ITT:** mPFS 18.89 vs 15.80 months, HR 0.65, 95% CI 0.48–0.89, *P* = 0.007; mDoR 27.10 vs 15.11 months, HR 0.57, 95% CI 0.36–0.91, *P* = 0.0181 **Patients chosen not to receive induction chemotherapy:** mPFS 21.72 vs 12.45 months, HR 0.55, 95% CI 0.34–0.88, *P* = 0.0111; **Patients chosen to receive induction chemotherapy:** mPFS 16.89 vs 16.85 months, HR 0.75, 95% CI 0.50–1.13, *P* = 0.1633; CBR 68.8% vs 67%; ORR 63.3% vs 55.7%	Grade ≥ 3 AE 50.4% vs 38.7%	[135]
**ALTERNATIVE** (Phase III) NCT01160211	1–2 lines	355 Postmenopausal only	Aromatase Inhibitor plus Trastuzumab(*n* = 117) **vs** Lapatinib(*n* = 118) **vs** Combination(*n* = 120)	mPFS 5.6 vs 8.3 vs 11 months, HR 0.85, 95% CI (0.45–0.88), *P* = 0.3159; HR 0.62, 95% CI (0.62–1.17), *P* = 0.0063 mOS 40 vs 45.1 vs 46 months, HR 0.85, 95% CI (0.45–0.88), *P* = 0.718; HR 0.62, 95% CI (0.62–1.17), *P* = 0.070 CBR 30% vs 34% vs 40%; ORR 13.7% vs 18.6% vs 31.7%	Grade ≥ 3 AE 10% vs 17% vs 14%; Discontinuation 6% vs 9% vs 3%; Cardiac events 3% vs 2% vs 7%; Declines in LVEF(> 20%) 2% vs 0% vs 3%	[136]
**PATRICIA** (Phase II) NCT02448420	2–4 prior lines	71 Postmenopausal only	Palbociclib plus Trastuzumab and Letrozole(*n* = 28) **vs** Trastuzumab(*n* = 28)	6-months PFS 46.4% vs 42.9%; mPFS 5.1 vs 6.0 months ORR 21.4% vs 14.3%; CBR 71.4% vs 75%	Grade ≥ 3 AE 92.9% vs 85.7%	[142]
**MonarcHER** (Phase II) NCT02675231	> 2 lines (TDM-1 and Paclitaxel treated)	237	Abemaciclib, Trastuzumab and Fulvestrant(*n* = 79) **vs** Abemaciclib and Trastuzumab(*n* = 79) **vs** Trastuzumab and single-agent CT(*n* = 79)	mPFS 8.3 vs 5.7 vs 5.7 months; HR 0.67, 95% CI 0.45–1.00, *P* = 0.051; HR 0.94, 95% CI 0.64–1.38, *P* = 0.769 mOS 31.1 vs 29.2 vs 20.7 months; HR 0.71, 95% CI 0.48–1.05, *P* = 0.086; HR 0.83, 95% CI 0.57–1.23, *P* = 0.365 ORR 36% vs 16% vs 16%; CBR 59% vs 47% vs 33%	Grade ≥ 3 AE 68% vs 50.4% vs 48.3%; Discontinuation 3% vs 1% vs 1%	[144]
**PLEASURABLE** (Phase II) NCT03772353	1–2 lines	48 Postmenopausal only	**Single arm:** Dalpiciclib and Pyrotinib and ET(letrozole or fulvestrant)	ORR 68.1%, DCR 100% *PFS and OS data were immature Trastuzumab-naive **vs** Trastuzumab-pretreated: ORR 81.3% (13/16) vs 61.3% (19/31) Previous Phase Ib study **LORDSHIPS**: ORR 66.7%, DCR 93.3%; mPFS 11.3 months	Grade ≥ 3 AE Leukopenia 45.8%, Neutropenia 66.7%, Anemia 4.2%, Thrombocytopenia 4.2%, Diarrhea 2.1%, oral mucostitis 2.1%	[146]
**AFT-38** **PATINA** (Phase III) NCT02947685	First line maintenance	518	Palbociclib plus anti-HER2 and ET **vs** Anti-HER2 and ET	mPFS 44.3 vs 29.1 months; HR 0.74, 95% CI 0.58–0.94, *P* = 0.0074; Anti-HER2 pretreated subgroup: HR 0.68, 95% CI 0.54–1.07 ORR: 29.9% vs 22.2%, *p* = 0.046; CBR: 89.3% vs 81.3% mOS NE vs 77 months; HR 0.86, 95% CI 0.60–1.24; 3y-OS: 87% vs 84.7%; 5y-OS: 74.3% vs 69	Grade ≥ 3 AE Neutropenia 67.8% vs 2%, Diarrhea 11.1% vs 1.6%;	[146]
**DETECT V** (Phase III) NCT02344472	1–3 lines	271	Trastuzumab and Pertuzumab plus ET (and Ribociclib) **vs** CT	**CT-free vs CT-containing:** mOS NR vs 46.1 months, HR 1.07, 95% CI 0.65–1.77, *p = *0.79; mPFS 19.1 vs 22.4 months, HR 1.19, 95% CI 0.84–1.69, *p = *0.34 **Ribociclib vs no ribociclib:** mOS NR vs 46.1 months, HR 0.42, 95% CI 0.24–0.74, *p* = 0.002; mPFS 27.2 vs 15.6 months, HR 0.52, 95% CI 0.37–0.75, *p* < 0.001	Grade ≥ 3 AE CT-free vs CT-containing: 54.8% vs 61%; Ribociclib vs no ribociclib: 64.6% vs 50%	[145]

The bold values is intended to highlight and differentiate the data between the Intention-to-Treat population and subgroup population

The PI3K/AKT/mTOR pathway's dysregulation contributes to both anti-HER2 and endocrine therapy resistance [11, 24]. A phase I study [147] of alpelisib(PI3Kα inhibitor) combined with T-DM1 received promising activity (ORR 43%, CBR 71%) in trastuzumab-resistant HER2+ MBC, including T-DM1-resistant cases (ORR 30%, CBR 60%). However, phase III BOLERO-1 [148] and BOLERO-3 [149] draw the same conclusions that everolimus(mTOR inhibitor) added to trastuzumab-based chemotherapy did not improve PFS, especially in the HR+ subpopulation. Exploratory analysis suggested potential benefit in PTEN-loss patients (HR, 0.35; 95% CI, 0.15 to 0.8) [150], while the BOLERO-2 trial [151] established everolimus's efficacy in AI-pretreated HR+ MBC (mPFS 7.8 vs 3.2 months, HR 0.45, 95% CI 0.38–0.54). Given PIK3CA mutations predict PI3K inhibitor response in HR+ disease [29], further investigation is warranted to determine whether dual ER/HER2 blockade combined with mTOR/PI3K inhibitors could benefit HR+/HER2+ patients.

## Future aspects and conclusions

Selecting eligible patients for treatment escalation or de-escalation based on individual tumor characteristics or intrinsic biology is a major challenge in TPBC patients. Practical tools are needed to identify patients who are sensitive to specific drugs. HER2DX, a multivariable prognostic score tool, has been widely used in clinical practice [152]. Retrospective diagnostic/prognostic analysis of the GOM-HGUGM-2018–05 study and combined analysis of two previously reported neoadjuvant cohort studies (DAPHNe and I-SPY2) have validated that the HER2DX pCR score correlates with the pCR probability of trastuzumab-based neoadjuvant therapy [153]. The PerELISA trial also demonstrated HER2DX’s effectiveness in predicting the response to neoadjuvant targeted therapy in HR+/HER2+ patients and its association with the Ki-67 response following 2 weeks of letrozole treatment [154].

Studies have shown that combining the HER2-enriched intrinsic subtype with ERBB2 mRNA levels can identify patients sensitive to HER2-targeted therapy [93]. PATRICIA trial found that the PAM50 Luminal subtype benefits most from CDK4/6i, significantly improving PFS (mPFS for Luminal 10.6 months vs. non-Luminal 4.2 months, *P* = 0.002) and achieving the highest ORR (luminal 21.7% months and non-luminal 14.3%, *P* = 0.525) [142]. Consistent with these findings, exploratory study from MonarcHER trial demonstrated that the Luminal subtype had better PFS and OS with additional abemaciclib (mPFS 8.6 vs 5.4 months, HR 0.54, 95% CI 0.38–0.79; mOS 31.7 vs 19.7 months, HR 0.68, 95% CI 0.46–1.00). In addition to PAM50 intrinsic subtypes, other approaches are exploring how to categorized patients into different suitable treatment regimen. The I-SPY2 trial, an adaptive trial of neoadjuvant therapy for high-risk BC, incorporates Immune, DNA repair, and HER2/Luminal phenotypes to better predict drug response [155]. Among HER2+ subtype, patients classified as BluePrint(BP)-Luminal are more responsive to ATK inhibitor MK2206 (pCR 60%), while BP-HER2/-Basal patients respond better to pertuzumab (pCR 78%). When focusing on receptor subtypes, the HR+/HER2+ patients were mostly responsive to T-DM1 plus pertuzumab (pCR 51%) [156]. Additionally, a cohort from Fudan Hospital identified a Luminal A-like subgroup in TPBC patients based on the protein expression of CDCA8, BCL2, and STC2. This subgroup was associated with a better prognosis but showed reduced benefit from trastuzumab, suggesting the potential for treatment de-escalation or endocrine-based therapies [41].

Future efforts in TPBC should focus on precision medicine by distinguishing the driver pathway or target gene to initiate suitable treatment therapy. Biology-driven trials are needed to investigate the efficacy of individual-designed therapy compared to current HER2 BC treatments.

## Data Availability

This study did not generate any new datasets. All data analyzed are from publicly available sources, as cited in the manuscript.
